# Prevalence and Predictors of Disability 24-Months after Injury for Hospitalised and Non-Hospitalised Participants: Results from a Longitudinal Cohort Study in New Zealand

**DOI:** 10.1371/journal.pone.0080194

**Published:** 2013-11-21

**Authors:** Sarah Derrett, Suzanne Wilson, Ari Samaranayaka, John Langley, Emma Wyeth, Shanthi Ameratunga, Rebbecca Lilley, Gabrielle Davie, Melbourne Mauiliu

**Affiliations:** 1 Injury Prevention Research Unit, Department of Preventive and Social Medicine, Dunedin School of Medicine, University of Otago, Dunedin, New Zealand; 2 Te Roopū Rangahau Hauora Māori a Ngāi Tahu (Ngāi Tahu Māori Health Research Unit), Department of Preventive and Social Medicine, Dunedin School of Medicine, University of Otago, Dunedin, New Zealand; 3 School of Population Health, Faculty of Medical and Health Sciences, University of Auckland, Auckland, New Zealand; 4 School of Health and Social Services, College of Health, Massey University, Palmerston North, New Zealand; Wake Forest University Health Sciences, United States of America

## Abstract

**Introduction:**

Most studies investigating disability outcomes following injury have examined hospitalised patients. It is not known whether variables associated with disability outcomes are similar for injured people who are not hospitalised.

**Aims:**

This paper compares the prevalence of disability 24 months after injury for participants in the Prospective Outcomes of Injury Study who were hospitalised and those non-hospitalised, and also seeks to identify pre-injury and injury-related predictors of disability among hospitalised and non-hospitalised participants.

**Methods:**

Participants, aged 18–64 years, were recruited from an injury claims register managed by New Zealand’s no-fault injury compensation insurer after referral by health care professionals. A wide range of pre-injury socio-demographic, health and psychosocial characteristics were collected, as well as injury-related characteristics; outcome is assessed using the WHODAS. Multivariable models estimating relative risks of disability for hospitalised and non-hospitalised participants were developed using Poisson regression methods.

**Results:**

Of 2856 participants, analyses were restricted to 2184 (76%) participants for whom both pre-injury and 24 month WHODAS data were available. Of these, 25% were hospitalised. In both hospitalised and non-hospitalised groups, 13% experience disability (WHODAS≥10) 24 months after injury; higher than pre-injury (5%). Of 28 predictor variables, seven independently placed injured participants in the hospitalised group at increased risk of disability 24 months after injury; eight in the non-hospitalised. Only four predictors (pre-injury disability, two or more pre-injury chronic conditions, pre-injury BMI≥30 and trouble accessing healthcare services) were common to both the hospitalised and non-hospitalised groups. There is some evidence to suggest that among the hospitalised group, Māori have higher risk of disability relative to non-Māori.

**Conclusions:**

At 24 months considerable disability is borne, equally, by hospitalised and non-hospitalised groups. However, predictors of disability are not necessarily consistent between the hospitalised and non-hospitalised groups, suggesting caution in generalising results from one group to the other.

## Introduction

Injuries are responsible for significant health burdens in terms of premature mortality from fatal injuries and disability to injury survivors. Recently, the Global Burden of Disease report from the World Health Organisation (WHO) revealed that reductions in the disability-adjusted life year (DALY) burden associated with communicable, maternal, neonatal, and nutritional disorders have been achieved, but that similar gains have not been demonstrated for injury [1]. Increasingly, the disability-related burden experienced by survivors of injury is the focus of attention from clinicians, policy-makers and researchers [2]–[5]. Nonetheless, studies investigating outcomes resulting from a wide range of injury types are limited. Where studies of injury outcome have been reported, they are often restricted to recruiting people via hospitals; few studies have reported outcomes for injured people not admitted to hospital due to their injury, and even fewer have used recognised measures of disability outcome [5]–[9].

We have previously reported the prevalence of disability outcomes three months after injury for participants in the Prospective Outcomes of Injury Study (POIS) underway in New Zealand [9]. Proportionately more participants reported disability three months after their injury than before it, both for those who were hospitalised (54% versus 5% respectively) and those who were not (39% versus 5% respectively). We also found that only three of the 27 variables included in the multivariable models – pre-injury disability, BMI≥30 and injury severity – were independently associated with increased odds of disability three months after injury for both the hospitalised and non-hospitalised groups; other variables were also associated with increased odds of disability, but only among either the hospitalised group or the non-hospitalised group [9].

In this paper, we investigate whether the difference in risk factors observed three months after injury remains when longer-term outcomes are considered. POIS was developed with careful attention paid to the principles of the Treaty of Waitangi, a treaty of cession signed by representatives of Māori (New Zealand’s indigenous population; Māori comprise 15% of the total New Zealand population) and the British Crown in 1840 [10], to ensure that disability outcomes for Māori could be better understood [9], [11]. Like many other indigenous populations throughout the world, Māori experience numerous health disparities compared to non-Māori; injury and disability are no exception. Despite concerns about health and disability disparities for Māori, and also Pacific peoples, having been identified within New Zealand, there is scant knowledge about injury-related disability outcomes for these populations [12], [13].

This paper compares the prevalence of disability 24 months after injury for POIS participants who were hospitalised and those who were non-hospitalised, especially for participants reporting Māori or Pacific ethnicity. The paper also seeks to identify predictors of participants’ disability in terms of their pre-injury socio-demographic, disability, health and psychosocial and injury-related characteristics, to help inform future development of appropriate interventions.

## Methods

The study was undertaken following approval from the New Zealand Health and Disability Multi-Region Ethics Committee (MEC/07/07/093). Following feedback from participants in the pilot study and to be inclusive of all people (including those with poor vision or limited literacy), and with the approval of the Ethics Committee, all participants granted oral consent to participate after receiving comprehensive information about the study. Oral consent was documented by interviewers, and all participants received copies of the consent form.

### Study Participants

The design of POIS and the main characteristics of participants have been described previously [11], [14]–[16]. In summary, potential participants were aged 18–64 years (inclusive) and lived in one of five geographical regions in New Zealand. Following referral by an accredited healthcare professional, participants had all been placed on an injury (entitlement claims) register at the Accident Compensation Corporation (ACC), New Zealand’s no-fault injury compensation insurer. Entitlement claimants have injuries serious enough to potentially require support, such as income compensation (if in paid employment), medical treatment and/or social and rehabilitation services; people with injuries resulting from self-harm or sexual assault were excluded from this study [14], [17]. ACC sent letters about the study to 7875 entitlement claimants on behalf of the research team; of these, 4881 people were subsequently able to be contacted by the POIS research team; 2856 (59%) participated in their first interview between December 2007 and August 2009 [15]. This paper uses data collected at interviews held three months (median = 3.2; interquartile range, IQR = 2.5,4.2) and 24 months (median = 24.4; IQR = 24.1, 25.1) after injury.

### Outcome

Disability outcome was measured using the brief WHO Disability Assessment Schedule (WHODAS II 12-item) [18]. Participants were asked to report WHODAS status over the 30 days before the 24 month interview. Scores are as described previously, with a possible range from 0–48 [9], [19]. Participants were grouped as having ‘disability’ if their WHODAS score was ≥10, and as having ‘no (or lesser) disability’ if their score was <10 [9]. At the three month interview, participants also reported their pre-injury WHODAS status for the 30 days prior to their injury, permitting adjustment of disability outcome for pre-injury disability.

### Hospitalisation

Data from POIS participants were probabilistically linked to a national database (the National Minimum Data Set) to identify participants admitted to hospital or treated at an Emergency Department for at least three hours, within seven days of the injury event, as described previously [9]. Those linked were classified as ‘hospitalised’ and those not as ‘non-hospitalised’.

### Explanatory Variables

Explanatory variables were grouped according to pre-injury socio-demographic, pre-injury health and psychosocial, and injury-related, characteristics. These items have been described before, and are therefore only briefly summarised here [9].

#### Pre-injury socio-demographic characteristics

Participants reported socio-demographic characteristics at the time of the first interview including: age, sex, ethnicity, highest educational qualification and living arrangements, based on questions from the 2006 New Zealand Census [20]. All participants who reported ethnicities were categorised as ‘yes’ or ‘no’ for each of the Māori and Pacific ethnicity variables. People were considered Māori if they reported Māori as any of their ethnicities, and Pacific if they reported a Pacific ethnicity. If a participant reported both Māori and one or more Pacific ethnicities they were classified as ‘yes’ for Māori ethnicity and also as ‘yes’ for Pacific ethnicity. Ethnicities classified as ‘Pacific’ have been described previously, and included participants of Samoan, Cook Island Māori, Tongan and Niuean ethnicities [21]. Highest educational qualification was grouped as ‘no qualifications’, ‘secondary school’ (high-school) level or ‘post-secondary school’ qualifications (where these took three months or more to obtain). ‘Living arrangements’ were grouped as living: ‘alone’, ‘with non-family’ or ‘with family’ (including partner/spouse). People working full-time (≥30 hours per week) or part-time (<30 hours per week) were classified as ‘working for pay’; the remaining as ‘not working for pay’ [22]. Household income was categorised as ‘adequate’ if participants reported ‘just enough’, ‘enough’ or ‘more than enough’ total pre-injury household income to meet their everyday needs; or ‘inadequate’ if they reported ‘not enough’ income [22].

#### Pre-injury health and psychosocial characteristics

Overall pre-injury ‘general health’ was rated on a five-point scale (‘excellent’, ‘very good’, ‘good’, ‘fair’ or ‘poor’) [23]. To assess pre-injury chronic conditions, participants reported whether they had been told by a doctor they had one, or more, of a list of 22 chronic illnesses or diseases (e.g. asthma, cancer, diabetes, depression or anxiety) that had lasted, or was expected to last, for more than six months [24]. Participants were defined as having a depressive-type episode if they responded affirmatively to either of two screening questions: that nearly every day, for a period of two weeks or more in the year before injury, they had felt ‘sad, blue or depressed’ or ‘loss of interest in things like work or hobbies or things they usually like to do for fun’ [25]. Participants who ‘strongly agreed’ or ‘agreed’ with a statement that “Overall, I expect more good things to happen to me than bad” were categorised as ‘optimistic’ and compared with the rest [26]. ‘Self-efficacy’ was based on the General Self-Efficacy Scale [27]. A score ≤25 was classified as poor self-efficacy [9]. A single question from FACIT-Sp (permission granted by www.facit.org) asked participants if they found “comfort in faith and spiritual beliefs” [28].

Pre-injury ‘family involvement’ was assessed by asking whether family played a ‘very large’, ‘large’, ‘small’ or ‘very small’ part in participants’ lives [22]. Participants rated their overall satisfaction with ‘social relationships’ [9]. Those reporting they were ‘completely’ or ‘mostly’ satisfied were classified as ‘satisfied’; those reporting being ‘neither satisfied nor dissatisfied’, ‘mostly’ or ‘completely’ dissatisfied were classified as ‘not satisfied’. ‘Sense of community’ was assessed by asking participants to state whether they felt their neighbourhood’s ‘sense of community’ was ‘strong’, ‘very little’ or ‘something in-between’ [29].

Pre-injury levels of physical activity were assessed by asking the number of days in the seven-day period prior to injury they had engaged in either 30 minutes of moderate activity (including brisk walking) or 15 minutes of vigorous activity [30]. ‘Sleep’ was assessed by asking the number of nights per week that they (usually) had seven or more hours sleep [9]. Body Mass Index (BMI) was categorised as BMI<30 and BMI≥30 [9], [31]. Pre-injury smoking was assessed by asking whether or not people smoke cigarettes regularly [20]. Participants were grouped into three ‘alcohol use’ categories according to their reported consumption in the year before injury using the brief Alcohol Use Disorders Identification Test (AUDIT-C) [9], [32]. Participants were also asked about ‘recreational drug use’ [9].

#### Injury-related characteristics

At the three month interview, participants were asked to report whether the injury cause was intentional (assault); whether, at the time of injury, they felt the injury was a threat to their life; and a threat of severe longer-term disability. For each of these separate variables, those responding ‘yes’ or ‘maybe/possibly’ were grouped together and compared to those responding ‘no’. Information about post-injury access to healthcare services was obtained by asking people at the three month interview if they had trouble getting to or contacting health services; ‘yes’ and ‘mixed’ were grouped together and compared to those who said ‘no’.

Twelve injury type (injury region/nature) variables, based on the International Classification of Diseases (ICD-10) injury mortality diagnosis matrix and the Barell injury diagnosis matrix, were developed using ACC data to describe the injured body region and nature of the injury [9], [33], [34]. All participants were classified ‘yes’ or ‘no’ for each variable according to whether or not they had sustained that injury type [35]. New Injury Severity Scores (NISS) were also derived for each participant and grouped into three categories: 1–3 (least severe), 4–6 and >6 (most severe) [9], [36].

### Analysis

Chi-squared tests were used to compare proportions hospitalised and non-hospitalised according to each explanatory variable. Proportions with a WHODAS score≥10 24 months after injury are presented with 95% confidence intervals (95%CI).

To identify possible predictors of disability 24 months after injury we used Poisson models with robust standard errors [37]. Using these models we can directly estimate the relative risks for binary outcomes. A two-part process was used for model-building. Firstly, independent models were built for each of the hospitalised and non-hospitalised sub-groups using a stepwise backward selection procedure with a p-value threshold of ≤0.10. All participants and all explanatory variables listed in Tables 1, 2, 3 were eligible for inclusion in this step; certain key variables (pre-injury WHODAS, age, sex, NISS, ethnicity, 12 injury types) were retained in all models; time between date of injury and 24 month interview was adjusted for in all models [9]. Prior knowledge and use of results from previous analyses informed identification of variables to be considered or retained, irrespective of p-values, to mitigate drawbacks associated with automated stepwise techniques available in statistical software. In our earlier paper, examining disability three months after injury [9], a separate ‘undisclosed’ category was created for three variables with high item-missingness (BMI, comfort in faith or spiritual beliefs and sense of community) to allow participants’ missing responses for these variables to be included in model-building. These ‘undisclosed’ categories were maintained in this analysis too.

**Table 1:** Pre-injury socio-demographic characteristics of 24-month interview participants according to hospitalisation status (N = 2184).

Characteristics		Hospitalised		Non-hospitalised		P value**
		n = 548	%*	n = 1636	%*	
Age	18–24 years	70	12.8	178	10.9	0.40
	25–34 years	111	20.3	321	19.6	
	35–44 years	125	22.8	366	22.4	
	45–54 years	127	23.2	444	27.1	
	55–64 years	115	21.0	327	20.0	
Sex	Male	358	65.3	923	56.4	<0.001
	Female	190	34.7	713	43.6	
Māori ethnicity#	No	442	80.8	1365	83.5	0.32
	Yes	105	19.2	269	16.5	
Pacific ethnicity#	No	503	92.0	1532	93.8	0.33
	Yes	44	8.0	102	6.2	
Highest educational qualification	Post-secondary school	331	61.1	1002	61.9	0.94
	Secondary school	132	24.4	384	23.7	
	No qualifications	79	14.6	234	14.4	
Living arrangements	With family	443	81.0	1356	83.2	0.39
	With non-family	43	7.9	123	7.6	
	Alone	61	11.2	150	9.2	
Working for pay	Yes	498	90.9	1518	92.8	0.13
	No	50	9.1	117	7.2	
Household income	Adequate	498	92.6	1481	91.0	0.27
	Inadequate	40	7.4	146	9.0	

* Column percentage. Missing cases excluded from numerator and denominator.

** P value from Chi-squared test to compare hospitalised and non-hospitalised groups for each factor considered.

#Multiple ethnicities possible.

**Table 2:** Pre-injury health and psychosocial characteristics of 24-month interview participants according to hospitalisation status (N = 2184).

Characteristics		Hospitalised		Non-hospitalised		P value**
		n = 548	%*	n = 1636	%*	
Pre-injury WHODAS score	0 to 9	521	95.1	1548	94.6	0.68
	≥10	27	4.9	88	5.4	
General health	Excellent/Very good/Good	524	95.8	1540	94.4	0.21
	Fair/Poor	23	4.2	91	5.6	
Chronic conditions	0	285	53.4	805	50.9	0.39
	1	148	27.7	435	27.5	
	≥2	101	18.9	342	21.6	
Depressive-type episode	No	446	81.4	1317	80.8	0.74
	Yes	102	18.6	314	19.3	
Optimism	Yes	496	91.3	1411	87.6	0.02
	No	47	8.7	200	12.4	
Self-efficacy	Not poor	506	93.0	1468	90.7	0.09
	Poor	38	7.0	151	9.3	
Comfort in faith or spiritual beliefs	Very much/Quite a bit	171	31.2	555	33.9	0.28
	Some/A little bit/None	359	65.5	1013	61.9	
	Undisclosed	18	3.3	68	4.2	
Family involvement	Very large/Large	482	88.3	1468	90.3	0.17
	Small/Very small	64	11.7	157	9.7	
Social relationships	Satisfied	513	94.1	1534	94.2	0.93
	Not satisfied	32	5.9	94	5.8	
Sense of community	Strong	149	27.2	493	30.1	0.63
	In-between	237	43.3	683	41.8	
	Little	136	24.8	385	23.5	
	Undisclosed	26	4.7	75	4.6	
Physical activity	≥5 days	300	56.0	863	53.7	0.37
	<5 days	236	44.0	743	46.3	
Sleep	≥5 nights	416	77.5	1221	75.8	0.43
	<5 nights	121	22.5	390	24.2	
BMI	<30	401	73.2	1180	72.1	0.37
	≥30	123	22.5	401	24.5	
	Undisclosed	24	4.4	55	3.4	
Smoking	No	405	74.2	1196	73.2	0.65
	Yes	141	25.8	438	26.8	
Alcohol use	Low	255	47.0	813	50.0	0.29
	Moderate	183	33.7	543	33.4	
	High	105	19.3	271	16.7	
Recreational drug use	No	444	81.3	1364	83.5	0.25
	Yes	102	18.7	270	16.5	

* Column percentage. Missing cases excluded from numerator and denominator unless labelled as Undisclosed.

** P value from Chi-squared test to compare hospitalised and non-hospitalised groups for each factor considered.

**Table 3:** Injury-related characteristics of 24-month interview participants according to hospitalisation status (N = 2184).

Characteristics		Hospitalised		Non-hospitalised		P value**
		n = 548	%*	n = 1636	%*	
Injury cause	Unintentional	514	94.1	1585	97.2	<0.001
	Intentional (assault)	32	5.9	45	2.8	
Threat to life	No	407	76.7	1498	92.4	<0.001
	Yes/Maybe	124	23.4	123	7.6	
Threat of severe long-term disability	No	257	48.3	1018	63.1	<0.001
	Yes/Maybe	275	51.7	596	36.9	
Access to healthcare services	No trouble	477	88.0	1471	90.9	0.05
	Trouble/mixed	65	12.0	148	9.1	
Injury severity	NISS 1–3	133	24.9	724	45.9	<0.001
	NISS 4–6	272	50.8	675	42.8	
	NISS >6	130	24.3	177	11.2	
*Injury types* #						
Intracranial injury	No	504	92.0	1595	97.5	<0.001
	Yes	44	8.0	41	2.5	
Head/neck superficial injury	No	506	92.3	1596	97.6	<0.001
	Yes	42	7.7	40	2.4	
Spine sprain or dislocation	No	509	92.9	1328	81.2	<0.001
	Yes	39	7.1	308	18.8	
Upper extremity fracture	No	398	72.6	1393	85.2	<0.001
	Yes	150	27.4	243	14.9	
Upper extremity sprain or dislocation	No	485	88.5	1393	85.2	0.05
	Yes	63	11.5	243	14.9	
Upper extremity open wound	No	495	90.3	1579	96.5	<0.001
	Yes	53	9.7	57	3.5	
Upper extremity superficial injury	No	524	95.6	1559	95.3	0.75
	Yes	24	4.4	77	4.7	
Lower extremity fracture	No	387	70.6	1419	86.7	<0.001
	Yes	161	29.4	217	13.3	
Lower extremity sprain or dislocation	No	475	86.7	1151	70.4	<0.001
	Yes	73	13.3	485	29.7	
Lower extremity open wound	No	508	92.7	1592	97.3	<0.001
	Yes	40	7.3	44	2.7	
Lower extremity superficial injury	No	515	94.0	1522	93.0	0.44
	Yes	33	6.0	114	7.0	
Other injury	No	377	68.8	1415	86.5	<0.001
	Yes	171	31.2	221	13.5	

* Column percentage. Missing cases excluded from numerator and denominator.

** P value from Chi-squared test to compare hospitalised and non-hospitalised groups for each factor considered.

#Multiple injury types possible.

All variables retained in either of the independent models for the hospitalised and non-hospitalised groups were then consistently retained in two further models, allowing us to present relative risks consistently for both groups. This was done to allow readers to ascertain whether or not a variable which is (say) marginally non-significant in the hospitalised group, may have an effect in the same direction as a significant finding in the non-hospitalised group. The final multivariable models (complete case analyses) include all participants with non-missing responses for the retained variables (apart from the three mentioned above with an ‘undisclosed’ category). Model fit was assessed using deviance goodness-of-fit test.

Missingness in our complete case analysis is unlikely be ‘missing completely at random’ [38], therefore results from this analysis may be biased. We undertook sensitivity analyses using inverse probability weighting to investigate this [39]. The main reason for participants not being included in the complete case analysis was not facing the 24 month interview. Our previous analyses identified males, young adults, Māori, participants living with non-family members, and those with inadequate household income as more likely to not participate in the 24 month interview [40]. Further to this, in our present study, by comparing participants with complete data with those with incomplete data, we identified missingness as being related to BMI, smoking, and depressive-type episode status. Therefore, we used each of the above variables as predictors in a logistic regression to estimate the probability of each participant being in the complete case analysis, and then re-analysed the complete cases using weights equal to the inverse of that probability. Results from this analysis were compared with those from the complete case analysis to investigate the sensitivity of our results to missingness (see Discussion).

Stata 12.1 was used for analyses [41].

## Results

Of 2856 participants in the three month interview, 2256 (79%) participated in the 24 month interview. As some were missing pre-injury or 24 month WHODAS disability outcome scores, the analyses are restricted to the 2184 (76%) participants for whom both pre-injury and 24 month follow-up WHODAS data were available. Of these, 548 (25%) were hospitalised within seven days of the injury event; the remainder were classified as non-hospitalised (n = 1636).

### Univariate Analyses

Tables 1, 2, 3 present pre-injury socio-demographic, pre-injury health and psychosocial, and injury-related characteristics according to whether or not participants had been hospitalised. Among pre-injury socio-demographic characteristics, a greater proportion of males were hospitalised (Table 1). Apart from pre-injury optimism which was reported more among those hospitalised, no statistically significant differences between the hospitalised and non-hospitalised groups were observed in proportions reporting pre-injury health and psychosocial characteristics, including pre-injury disability (WHODAS≥10) (Table 2). Differences are apparent between the groups for many of the injury-related characteristics (Table 3). Intentional injury cause (assault) was more prevalent among the hospitalised, as was a perceived threat to their life at the time of injury, a threat of longer-term disability and injury severity scores of NISS≥4. Seven injury type variables were more prevalent among the hospitalised group, and two (spine and lower extremity sprain/strain or dislocation) more prevalent among the non-hospitalised.

Similar proportions in both the hospitalised and non-hospitalised groups, were experiencing disability (WHODAS≥10) at 24 months (13.1%;95%CI = 11.4%,14.7% and 13.0%;95%CI = 10.4%,16.3% respectively). There were no substantial differences in 24 month disability according to age or sex for either the hospitalised or non-hospitalised groups (Table 4). For other pre-injury socio-demographic variables such as education, working for pay and household income, differences in proportions experiencing disability are apparent among both the hospitalised and non-hospitalised groups, although 95%CI are not always distinct. For Māori, and considering the hospitalised and non-hospitalised groups together, the overall proportion experiencing disability 24 months after injury is 19%; and 15% for Pacific participants. The proportion of Māori experiencing disability was significantly higher than non-Māori for those hospitalised. Participants with pre-injury disability were more likely to have disability 24 months after injury than those with no/lesser pre-injury disability for both the hospitalised and non-hospitalised groups (Table 5). Among other pre-injury health and psychosocial variables, differences in proportions experiencing disability at 24 months can also be observed, but in most instances the 95%CI are not distinct. An exception is general health, among both the hospitalised and non-hospitalised groups, where a higher proportion of those with fair/poor health pre-injury experience disability 24 months after injury. A greater proportion of those with two or more pre-injury chronic conditions also experience disability 24 months after injury for both the hospitalised and non-hospitalised groups, as do those with a pre-injury depressive-type episode and those who reported smoking pre-injury. A greater proportion of those with BMI≥30 experience 24 month disability among the non-hospitalised group, as do those reporting less optimism pre-injury and not satisfied with social relationships pre-injury. A greater proportion of the hospitalised group experience 24 month disability if they reported poor self-efficacy or fewer nights with at least seven hours sleep pre-injury. Among the injury-related variables and considering the hospitalised group, higher proportions of those perceiving a threat to their life or a threat of disability experience disability at 24 months (Table 6). For the non-hospitalised group, higher proportions of those experiencing intentional injury cause (assault), experiencing post-injury trouble accessing healthcare services, sustaining an intracranial injury or spine sprain/dislocation, experience disability at 24 months; whereas smaller proportions with an upper extremity fracture experience disability.

**Table 4:** Prevalence of participants with disability (WHODAS≥10) 24 months after injury according to pre-injury socio-demographic characteristics and hospitalisation status.

Characteristics		Prevalence (95%CI) of WHODAS≥10 at 24-months					
		Hospitalised			Non-hospitalised		
		(n = 548)			(n = 1636)		
		%	95% CI		%	95% CI	
Age	18–24 years	5.7	1.6	14.0	10.1	6.1	15.5
	25–34 years	10.8	5.7	18.1	10.6	7.4	14.5
	35–44 years	15.2	9.4	22.7	10.4	7.4	14.0
	45–54 years	11.8	6.7	18.7	16.9	13.5	20.7
	55–64 years	19.1	12.4	27.5	14.4	10.8	18.6
Sex	Male	13.4	10.1	17.4	12.1	10.1	14.4
	Female	12.6	8.3	18.2	14.0	11.6	16.8
Māori ethnicity#	No	10.2	7.5	13.4	12.4	10.7	14.2
	Yes	25.7	17.7	35.2	16.0	11.8	20.9
Pacific ethnicity#	No	12.9	10.1	16.2	12.9	11.2	14.6
	Yes	15.9	6.6	30.1	14.7	8.5	23.1
Highest educational qualification	Post-secondary school	10.6	7.5	14.4	13.1	11.0	15.3
	Secondary school	14.4	8.9	21.6	9.9	7.1	13.3
	No qualifications	22.8	14.1	33.6	18.4	13.6	23.9
Living arrangements	With family	12.4	9.5	15.9	13.0	11.2	14.9
	With non-family	9.5	2.7	22.6	8.9	4.5	15.4
	Alone	21.3	11.9	33.7	16.0	10.5	22.9
Working for pay	Yes	11.2	8.6	14.4	12.5	10.8	14.2
	No	32.0	19.5	46.7	18.8	12.2	27.1
Household income	Adequate	11.8	9.1	15.0	12.6	10.9	14.4
	Inadequate	27.5	14.6	43.9	17.8	12.0	25.0

#Multiple ethnicities possible.

**Table 5:** Prevalence of participants with disability (WHODAS≥10) 24 months after injury according to pre-injury health and psychosocial characteristics and hospitalisation status.

Characteristics				Prevalence (95%CI) of WHODAS≥10 at 24-months					
				Hospitalised			Non-hospitalised		
				(n = 548)			(n = 1636)		
				%	95% CI		%	95% CI	
Pre-injury WHODAS score	0 to 9			10.7	8.2	13.7	11.3	9.8	13.0
	≥10			59.3	38.8	77.6	42.0	31.6	53.0
General health	Excellent/Very good/Good			12.0	9.4	15.1	11.8	10.2	13.5
	Fair/Poor			39.1	19.7	61.5	34.1	24.5	44.7
Chronic conditions	0			7.7	4.9	11.5	9.8	7.8	12.1
	1			10.1	5.8	16.2	11.5	8.7	14.9
	≥2			32.7	23.7	42.7	22.8	18.5	27.6
Depressive-type episode	No			11.4	8.6	14.8	10.9	9.3	12.7
	Yes			20.6	13.2	29.7	21.7	17.2	26.6
Optimism	Yes			12.3	9.5	15.5	12.3	10.6	14.1
	No			23.4	12.3	38.0	19.5	14.2	25.7
Self-efficacy	Not poor			12.1	9.3	15.2	12.5	10.9	14.3
	Poor			28.9	15.4	45.9	18.5	12.7	25.7
Comfort in faith or spiritualbeliefs	Very much/Quite a bit			15.2	10.2	21.5	14.8	11.9	18.0
	Some/A little bit/None			12.5	9.3	16.4	12.0	10.1	14.2
	Undisclosed			5.6	0.1	27.3	11.8	5.2	21.8
Family involvement	Very large/Large			12.4	9.6	15.7	12.7	11.0	14.5
	Small/Very small			18.8	10.1	30.5	15.3	10.0	21.9
Social relationships pre-injury	Satisfied			12.9	10.1	16.1	12.3	10.7	14.1
	Not satisfied			18.8	7.2	36.4	23.4	15.3	33.3
Sense of community	Strong			15.4	10.0	22.3	12.2	9.4	15.4
	In-between			11.8	8.0	16.6	11.1	8.9	13.7
	Little			12.5	7.5	19.3	15.8	12.3	19.9
	Undisclosed			15.4	4.4	34.9	20.0	11.6	30.8
Physical activity	≥5 days			13.7	10.0	18.1	12.4	10.3	14.8
	<5 days			12.3	8.4	17.2	13.7	11.3	16.4
Sleep	≥5 nights			10.6	7.8	13.9	12.7	10.9	14.7
	<5 nights			22.3	15.2	30.8	13.8	10.6	17.7
BMI	<30			10.7	7.9	14.2	11.3	9.5	13.2
	≥30			19.5	12.9	27.6	16.7	13.2	20.7
	Undisclosed			20.8	7.1	42.2	21.8	11.8	35.0
Smoking	No			10.9	8.0	14.3	11.5	9.8	13.5
	Yes			19.9	13.6	27.4	16.9	13.5	20.7
Alcohol use	Low			12.9	9.1	17.7	14.8	12.4	17.4
	Moderate			12.6	8.1	18.3	10.7	8.2	13.6
	High			15.2	9.0	23.6	11.8	8.2	16.3
Recreational drug use	No			11.9	9.1	15.3	13.3	11.5	15.2
	Yes			18.6	11.6	27.6	11.5	7.9	15.9

**Table 6:** Prevalence of participants with disability (WHODAS≥10) 24 months after injury according to pre-injury characteristics and hospitalisation status.

Characteristics		Prevalence (95%CI) of WHODAS≥10 at 24-months					
		Hospitalised			Non-hospitalised		
		(n = 548)			(n = 1636)		
		%	95% CI		%	95% CI	
Injury cause	Unintentional	12.8	10.1	16.0	12.5	10.9	14.2
	Intentional (assault)	18.8	7.2	36.4	31.1	18.2	46.6
Threat to life	No	10.8	8.0	14.2	12.6	10.9	14.3
	Yes/Maybe	20.2	13.5	28.3	18.7	12.2	26.7
Threat of severe long-term disability	No	6.6	3.9	10.4	12.5	10.5	14.7
	Yes/Maybe	19.3	14.8	24.4	13.9	11.2	17.0
Access to healthcare services	No trouble	11.9	9.2	15.2	12.2	10.5	13.9
	Trouble/Mixed	20.0	11.1	31.8	20.9	14.7	28.4
Injury severity	NISS 1–3	15.0	9.4	22.3	14.4	11.9	17.1
	NISS 4–6	9.6	6.3	13.7	11.0	8.7	13.6
	NISS >6	19.2	12.8	27.1	14.7	9.8	20.8
*Injury types#*							
Intracranial injury	No	12.5	9.7	15.7	12.5	11.0	14.3
	Yes	20.5	9.8	35.3	29.3	16.1	45.6
Head/neck superficial injury	No	12.8	10.1	16.1	12.7	11.1	14.4
	Yes	16.7	7.0	31.3	25.0	12.7	41.2
Spine sprain or dislocation	No	10.4	7.9	13.4	11.4	9.8	13.3
	Yes	23.1	11.1	39.3	19.5	15.2	24.4
Upper extremity fracture	No	14.3	11.0	18.2	13.9	12.1	15.8
	Yes	10.0	5.7	16.0	7.8	4.8	11.9
Upper extremity sprain or dislocation	No	13.2	10.3	16.5	12.3	10.6	14.1
	Yes	12.7	5.6	23.5	16.9	12.4	22.2
Upper extremity open wound	No	13.9	11.0	17.3	12.8	11.2	14.5
	Yes	5.7	1.2	15.7	17.5	8.7	29.9
Upper extremity superficial injury	No	12.8	10.0	16.0	12.7	11.1	14.5
	Yes	20.8	7.1	42.2	18.2	10.3	28.6
Lower extremity fracture	No	12.4	9.3	16.1	13.6	11.9	15.5
	Yes	14.9	9.8	21.4	8.8	5.4	13.3
Lower extremity sprain or dislocation	No	12.4	9.6	15.7	13.8	11.9	15.9
	Yes	17.8	9.8	28.5	10.9	8.3	14.0
Lower extremity open wound	No	14.0	11.1	17.3	13.1	11.5	14.9
	Yes	2.5	0.1	13.2	6.8	1.4	18.7
Lower extremity superficial injury	No	13.2	10.4	16.4	13.2	11.5	15.0
	Yes	12.1	3.4	28.2	9.6	4.9	16.6
Other injury	No	11.1	8.1	14.8	12.6	10.9	14.4
	Yes	17.5	12.2	24.1	15.4	10.9	20.8

#Multiple injury types possible.

### Multivariable Analyses

Table 7 presents the final multivariable models providing relative risks of disability 24 months after injury for both the hospitalised and non-hospitalised groups. In addition to the ‘undisclosed’ category created for three variables (BMI, comfort in faith or spiritual beliefs and sense of community), some participants were missing responses to one or more of the other variables in the final multivariable models. Data were available for 1964 (90%) of the 2184 participants in this analysis; 25% (n = 501) of these were hospitalised and 75% (n = 1463) non-hospitalised. Model fit was acceptable for both models (p = 0.98 and 0.94 for non-hospitalised and hospitalised respectively).

**Table 7:** Multivariable analyses of pre-injury and injury-related characteristics associated with disability (WHODAS≥10) 24 months after injury for hospitalised and non-hospitalised groups.

Characteristics		Hospitalised			Non-hospitalised		
		(n = 501)			(n = 1463)		
		RR*	95% CI		RR	95% CI	
**Pre-injury socio-demographic**							
Age	18–24 years	Ref			Ref		
	25–34 years	2.18	0.50	9.51	0.78	0.46	1.31
	35–44 years	3.96	0.91	17.20	0.78	0.46	1.32
	45–54 years	2.94	0.64	13.47	1.35	0.83	2.20
	55–64 years	2.72	0.57	13.04	1.12	0.67	1.89
Sex	Male	Ref			Ref		
	Female	0.98	0.57	1.72	1.03	0.79	1.34
Māori ethnicity	No	Ref			Ref		
	Yes	1.69	0.98	2.93	1.04	0.76	1.44
Pacific ethnicity	No	Ref			Ref		
	Yes	1.20	0.53	2.75	1.18	0.70	1.99
Living arrangements	With family	Ref			Ref		
	With non-family	0.58	0.23	1.49	0.73	0.42	1.30
	Alone	1.46	0.76	2.80	1.18	0.77	1.80
**Pre-injury health and psychosocial**							
Pre-injury WHODAS score	0–9	Ref			Ref		
	≥10	2.41	1.28	4.53	2.57	1.81	3.66
Chronic conditions	0	Ref			Ref		
	1	1.52	0.85	2.74	0.93	0.66	1.30
	≥2	3.02	1.57	5.78	1.42	1.02	1.98
Optimism	Yes	Ref			Ref		
	No	1.87	1.02	3.43	1.15	0.82	1.63
Depressive-type episode	No	Ref			Ref		
	Yes	0.72	0.40	1.29	1.39	1.04	1.88
Sense of community	Strong	Ref			Ref		
	In-between	0.99	0.60	1.64	0.88	0.64	1.21
	Little	0.79	0.44	1.41	1.25	0.90	1.73
	Undisclosed	1.44	0.51	4.06	1.38	0.76	2.51
BMI	<30	Ref			Ref		
	≥30	1.88	1.11	3.20	1.43	1.07	1.91
	Undisclosed	2.54	0.89	7.26	1.73	0.90	3.30
Smoking	No	Ref			Ref		
	Yes	1.56	0.97	2.52	1.31	0.99	1.73
**Injury-related**							
Injury cause	Unintentional	Ref			Ref		
	Intentional (assault)	0.87	0.36	2.12	2.49	1.38	4.49
Threat of severe long-term disability	No	Ref			Ref		
	Yes/Maybe	2.84	1.61	4.99	0.86	0.66	1.14
Access to healthcare services	No trouble	Ref			Ref		
	Trouble/Mixed	1.92	1.08	3.40	1.73	1.20	2.50
Injury severity	NISS 1–3	Ref			Ref		
	NISS 4–6	0.51	0.24	1.06	1.11	0.77	1.61
	NISS >6	0.84	0.37	1.90	1.23	0.79	1.93
*Injury types*							
Intracranial	No	Ref			Ref		
	Yes	1.41	0.62	3.19	2.03	1.03	4.04
Head/neck superficial	No	Ref			Ref		
	Yes	2.29	1.18	4.45	0.84	0.38	1.86
Spine sprain or dislocation	No	Ref			Ref		
	Yes	1.06	0.48	2.34	1.60	1.10	2.32
Upper extremity fracture	No	Ref			Ref		
	Yes	1.13	0.60	2.13	0.61	0.35	1.06
Upper extremity sprain or dislocation	No	Ref			Ref		
	Yes	1.14	0.46	2.82	1.40	0.95	2.06
Upper extremity open wound	No	Ref			Ref		
	Yes	0.43	0.12	1.57	1.47	0.82	2.63
Upper extremity superficial injury	No	Ref			Ref		
	Yes	1.34	0.54	3.33	1.43	0.81	2.53
Lower extremity fracture	No	Ref			Ref		
	Yes	1.35	0.72	2.54	0.86	0.50	1.49
Lower extremity sprain or dislocation	No	Ref			Ref		
	Yes	0.95	0.55	1.65	0.95	0.62	1.45
Lower extremity open wound	No	Ref			Ref		
	Yes	0.15	0.03	0.88	0.45	0.13	1.57
Lower extremity superficial injury	No	Ref			Ref		
	Yes	0.84	0.35	2.00	0.65	0.37	1.14
Other injury	No	Ref			Ref		
	Yes	1.30	0.77	2.18	1.32	0.87	2.00

* RR = Relative risk.

#### Pre-injury socio-demographic characteristics

Māori in the hospitalised group were at 70% increased risk of disability compared to non-Māori, whilst taking account of a range of pre-injury and injury-related variables in the modelling, although the 95%CI included 1 (95%CI = 1.0,2.9; p = 0.06). There was weak evidence (p = 0.02) to suggest risk of disability differed by age for those in the non-hospitalised group. There was no evidence to suggest that risk of disability differed for those within the separate categories of sex, Pacific ethnicity or living arrangements. Highest educational qualifications, working for pay and household income were not retained in the final models.

#### Pre-injury health and psychosocial characteristics

Having a pre-injury WHODAS≥10 increased the risk of post-injury disability (24 months after injury) for both the hospitalised (RR = 2.4; 95%CI = 1.3,4.5; p = 0.006) and non-hospitalised (RR = 2.6; 95%CI = 1.8,3.7; p<0.001) groups. Having two or more chronic conditions pre-injury, compared to having none, also independently predicted an increased risk of disability 24 months after injury among the hospitalised (RR = 3.0; 95%CI = 1.6,5.8; p = 0.001); and non-hospitalised (RR = 1.4; 95%CI = 1.0,2.0; p = 0.04) groups. Not being optimistic pre-injury increased the risk of disability among the hospitalised group (RR = 1.9; 95%CI = 1.0,3.4; p = 0.04); whereas having a depressive-type episode pre-injury increased the risk of disability among the non-hospitalised group (RR = 1.4; 95%CI = 1.0,1.9; p = 0.03). Having a BMI≥30 independently predicted an increased risk of disability compared to those with BMI<30 in both the hospitalised and non-hospitalised groups (RR = 1.9; 95%CI = 1.1,3.2; p = 0.02 and RR = 1.4; 95%CI = 1.1,1.9; p = 0.02, respectively). There was weak evidence that cigarette smoking was associated with an increased risk of disability among the hospitalised and non-hospitalised groups (RR = 1.6; 95%CI = 1.0,2.5; p = 0.07 and RR = 1.3; 95%CI = 1.0,1.7; p = 0.06, respectively). There was no evidence to suggest a relationship existed between sense of community and risk of disability. General health, self-efficacy, comfort in faith or spiritual beliefs, family involvement, social relationships, physical activity, sleep, and alcohol and recreational drug use were not retained in the final models.

#### Injury-related characteristics

Intentional injury cause (assault) increased the risk of disability among the non-hospitalised group only (RR = 2.5;95%CI = 1.4,4.5; p = 0.002). Perceived threat of longer-term disability at the time of injury increased the risk of disability among the hospitalised group only (RR = 2.8;95%CI = 1.6,5.0;p<0.001). Having trouble accessing healthcare services independently predicted an increased risk of disability for both the hospitalised and non-hospitalised groups (RR = 1.9;95%CI = 1.1,3.4;p = 0.03; RR = 1.7; 95%CI = 1.2,2.5; p = 0.003, respectively). Perceived threat to life at the time of the injury event was not retained in the final models.

There was no evidence to suggest a relationship existed between anatomical severity of injury and risk of disability for either the hospitalised or non-hospitalised group. For the hospitalised group head/neck superficial injury predicted risk of disability (RR = 2.3;95%CI = 1.2,4.4; p = 0.01); whereas intracranial injury (RR = 2.0; 95%CI = 1.0,4.0; p = 0.04) and spine sprain or dislocation (RR = 1.6;95%CI = 1.1,2.3;p = 0.01) predicted an increased risk of disability 24 months later among the non-hospitalised group. In the hospitalised group, those with a lower extremity open wound were at decreased risk of disability at 24 months compared to those with other injuries (RR = 0.15; 95%CI = 0.03,0.88; p = 0.04). There was insufficient evidence for the remaining injury types independently predicting risk of disability.

## Discussion

Our focus was on comparing hospitalised and non-hospitalised groups within a cohort comprising participants with a range of injury types, across a wide range of possible pre-injury and injury-related predictors of disability 24 months after injury (Tables 1, 2, 3, 4, 5, 6). Previously, we have reported that, three months after injury, the prevalence of disability was 54% for the hospitalised group and 39% for the non-hospitalised group [9]. By 24 months after injury, the prevalence of disability is considerably lower, at 13% for both. It is important to note that, while the proportion disabled has reduced over time, the reported pre-injury prevalence of disability (5%) has not been reached 24 months after injury [9]. Univariate analyses indicate that, of those hospitalised following injury, a greater proportion of Māori experience disability at 24 months than non-Māori (26% Māori compared to 10% non-Māori; p<0.001) (Table 4). Disability 24 months post-injury was also more prevalent among Māori than non-Māori for the non-hospitalised group, and among Pacific than non-Pacific peoples for both the hospitalised and non-hospitalised groups, but none of these differences were statistically significant.

Multivariable analyses indicate that pre-injury disability exposes injured participants to increased risk of disability 24 months later, regardless of whether or not they were hospitalised. Data from a large survey in the United States found that people with pre-existing disability face barriers to access to services [42]. However, in our study all participants had to have contact with at least one health provider to become registered with the no-fault compensation insurer (ACC); perhaps disparities in access occur post-registration. Māori who were hospitalised have 70% increased risk of disability 24 months after injury relative to hospitalised non-Māori. This result is of borderline statistical significance however, as with all other multivariable results, is found after controlling for differences in levels of pre-injury disability and other explanatory factors. ACC has previously identified that Māori are not gaining equitable access to ACC services [43]. As stated, all POIS participants had to have gained access to at least some ACC services to be recruited to POIS, and the multivariable model also included post-injury trouble accessing health care services. Disparities in access to certain treatments have been identified for Māori with other health conditions, even when they have gained access to the healthcare system [44]. Despite healthcare services increasingly recognising and incorporating Māori needs and values, perhaps there is still more work to be done in this area [45]. If our results are confirmed by others, they suggest that more attention needs to be focused on the post-injury treatment and rehabilitation processes for Māori to redress what appears to be a considerable disparity in outcome.

The analytic approach in this paper differs somewhat from the earlier three month analyses [9]. For example, here we estimate relative risks of disability outcome rather than the odds of disability and, although the range of possible explanatory factors is held constant between the earlier investigation and this one, Māori and Pacific ethnicities were added as specific variables here; factors retained in the final models also differ between the three month and 24 month analyses. Nevertheless, as in the 24 month analyses reported here, our three month paper revealed that few explanatory factors were consistently associated with an increased risk of disability across both the hospitalised and non-hospitalised groups; in fact only pre-injury disability, BMI≥30 and higher anatomical injury severity (NISS) were consistently associated across the two groups three months after injury [9]. By 24 months after injury, pre-injury disability and BMI≥30 again place people at increased risk of disability among both the hospitalised and non-hospitalised groups, but NISS is not independently associated with increased risk of disability in either the hospitalised or non-hospitalised group. The mechanism underlying the risk for injured people with BMI≥30 remains to be understood (and replicated in other studies); however, a meta-analysis has reported that obesity places people at increased risk of exit from work onto a disability pension [46]. At three months, post-injury trouble accessing healthcare services was associated with disability for the non-hospitalised group only [9]. By 24 months, trouble accessing healthcare services increased the risk of disability for both those with early hospital treatment and those without, relative to those reporting no trouble accessing healthcare services, and independently of age, sex, ethnicity, NISS and other potential explanatory factors. We cannot ascertain the precise mechanism underlying this increased risk of disability, but our findings suggest perceiving trouble accessing services may serve as a flag for the risk of longer-term disability. Likewise, at three months having two or more chronic conditions pre-injury was associated with disability for the hospitalised group only [9]. At 24 months, and again independently of pre-injury disability and the other variables in the models, those with two or more chronic conditions pre-injury had three times the risk of disability relative to those reporting no pre-injury chronic conditions in the hospitalised group and 1.4 times the risk in the non-hospitalised group.

As in our earlier paper, several factors were associated with disability outcome in one group or the other, but not in both [9]. Of 28 predictor variables included in the models, we identified seven that independently placed injured people at increased risk of disability 24 months after injury among the hospitalised group, and eight among the non-hospitalised group. As discussed above, four of these variables (pre-injury disability, having two or more pre-injury chronic conditions, pre-injury BMI≥30 and post-injury trouble accessing healthcare services) were common to both the hospitalised and non-hospitalised groups. Of those factors predicting risk of disability in one group only, those perceiving a threat of longer-term disability at the time of the injury event among the hospitalised group had the highest relative risk (nearly three-fold); the risk was not apparent for the non-hospitalised group. This finding suggests that asking people treated in hospital for their injury to report their perception of disability risk may well prove a useful indicator of those likely to experience disability outcomes in the longer-term. A lack of optimism pre-injury almost doubled the risk of 24 month disability among the hospitalised group only, as did head/neck superficial injury; whereas those with lower extremity open wound injuries who were hospitalised were at reduced risk of 24 month disability. Among the non-hospitalised group only, pre-injury depressive-type episode intentional cause (assault) and two of the 12 injury type variables (intracranial and spine sprain/dislocation) independently predicted increased risk of disability. Of particular note is intracranial injury among the non-hospitalised group that appears to have twice the risk of disability relative to those not having an intracranial injury.

Studies of injury outcome, including for people with a range of injury types, are being undertaken in different countries. As previously mentioned, finding studies directly comparable with ours is problematic due to the use of different outcome measures, variable follow-up rates, and different methods and recruitment strategies [3]. For example, the longitudinal UK Burden of Injury study recruited those attending emergency departments or admitted to hospital as a consequence of injury [5]. They have reported factors associated with self-reported recovery from injury and found, 12 months after injury, those aged 45–64 years, admitted to hospital and having a moderate or severe injury (approximating our NISS 4–6 and >6 categories) were at reduced risk of self-reported recovery relative to their reference groups [5]. Clearly, their study and outcome are not directly comparable to ours. Nor is the longitudinal study, undertaken in Norway, which identified low injury severity, not having a serious head injury, low levels of pre-injury depression, and being optimistic as independent predictors of return to work 12 months after injury among a cohort of injured patients aged 18–65 years recruited via a trauma centre [47]. A study from Norway, of 101 trauma patients with injury severity NISS≥16, has investigated post-injury factors associated with 24 month disability according to the WHODAS (36-item version) [48]. They found, as we did, that the proportions reporting WHODAS disability decreased with time, but not necessarily to the level reported in the general population (their study) or to pre-injury prevalence (our study). They found that gender was not associated with 24 month disability. Previously we reported women were more likely to experience disability three months after injury than men; others have also found being female places women at risk of poor outcomes [9], [49], [50]. However, as with the Norwegian study, we found women were not at increased risk of disability in the longer-term relative to men [48]. Again, more work is required to understand this finding.

### Strengths and Limitations

Strengths of our investigation include being able to recruit both those with an injury resulting in treatment at hospital and those not receiving hospital treatment, using a measure of outcome specifically developed to measure disability (WHODAS), being able to recruit participants with ‘all-injury’ types, and having a wide range of pre-injury and injury-related factors to include in our analyses. It is also a strength of our study that we were able to interview participants independently of ACC (New Zealand’s no-fault compensation insurer), thereby reducing the likelihood of perverse incentives leading to an exaggeration of poor outcome (e.g. in some litigious insurance systems participants may feel the impact of the injury needs to be sustained until their case has reached court or otherwise been resolved). In fact, possibly the very existence of New Zealand’s no-fault insurer reduces such incentives. Regardless, our participants were all interviewed independently of the insurer, and knew that their results were confidential to the university research team.

Our limitations include asking participants to recall a number of pre-injury states three months after the injury; although subsequent analyses of our cohort suggest that recall bias is likely to be minor, at worst [51]. Another limitation is that few participants had high NISS (none with NISS>22). However, a study undertaken in Denmark reported no associations between higher injury severity and long-term health-related quality of life outcomes [52].

Our multivariable model presented in Table 7 was a complete case analysis, based on 69% of the cohort. The Inverse Probability Weighted sensitivity analyses we conducted did not substantially alter the results in Table 7 in terms of variables that are significantly associated with disability at 24 months. However, the results in Table 7 may overestimate the relative risks for pre-injury disability, intracranial injury and assault by 4% to 7%, while underestimating the relative risks for ‘NISS 4–6’, upper extremity fractures, and lower extremity sprain or dislocation by 7 to 8% among the non-hospitalised group (results not presented). For the hospitalised group, Table 7 may overestimate the relative risks for BMI≥30, and 2 or more chronic conditions by 5 to 7%, and the effect of ‘NISS 4–6’ by 19%, while underestimating the effect of pre-injury disability, head/neck superficial injury, and trouble accessing health services by 10 to 15%. The relative risk of disability at 24 months for Māori in the hospitalised group may also be underestimated by 5%. Lastly, another limitation concerns a possible lack of precision accompanying some of our risk predictions where, in reality, risks may exist. For example, we may have lacked sufficient sample size to identify relationships between Pacific ethnicity and risk of disability at 24 months, or between other potential explanatory factors and disability where the numbers were small in some categories; particularly for the smaller hospitalised group.

### Conclusions

This study reports relationships between a wide range of pre-injury and injury-related variables and risk of longer-term (24 month) disability. Certain pre-injury and injury-related variables independently predict longer-term disability, but only four (pre-injury disability, having two or more pre-injury chronic conditions, pre-injury BMI≥30 and post-injury trouble accessing healthcare services) are common to both the hospitalised and non-hospitalised groups. As our earlier paper suggested, our results indicate that it may be unwise to generalise from results about predictors of risk for hospitalised patients to injured non-hospitalised groups [9]. The proportions experiencing disability at 24 months have reduced from the proportions experiencing disability at three months; but they have not reduced to pre-injury levels. A considerable disability burden continues to be borne, equally, by both the hospitalised and non-hospitalised groups. It is of particular concern that Māori may experience a higher risk of disability than non-Māori. Further analysis is planned to examine pre-injury and injury-related predictors of longer-term disability, specifically for Māori in our study, to investigate, in further detail, drivers of this increased risk. It will also be interesting to see if our findings are replicated in other studies. If so, and depending on the results of robust trials, it is to be hoped that identifying and implementing appropriate interventions aimed at improving outcomes for groups of people at increased risk may result in reduced post-injury disability.
